# Optimal Attenuation of Experimental Autoimmune Encephalomyelitis by Intravenous Immunoglobulin Requires an Intact Interleukin-11 Receptor

**DOI:** 10.1371/journal.pone.0101947

**Published:** 2014-07-31

**Authors:** Carlyn A. Figueiredo, Paulina C. Drohomyrecky, Stephen D. S. McCarthy, Danila Leontyev, Xue-Zhong Ma, Donald R. Branch, Shannon E. Dunn

**Affiliations:** 1 Research & Development, Canadian Blood Services, 67 College Toronto, Toronto, Ontario, Canada; 2 Departments of Laboratory Medicine and Pathobiology, University Health Network, Toronto, Ontario, Canada; 3 Toronto General Research Institute, University Health Network, Toronto, Ontario, Canada; 4 Department of Immunology, University of Toronto, and Centre for Innovation, Canadian Blood Services, Toronto, Ontario, Canada; 5 Women’s College Research Institute, Toronto, Ontario, Canada; University of Düsseldorf, Germany

## Abstract

**Background:**

Intravenous immunoglobulin (IVIg) has been used to treat a variety of autoimmune disorders including multiple sclerosis (MS); however its mechanism of action remains elusive. Recent work has shown that interleukin-11 (IL-11) mRNAs are upregulated by IVIg in MS patient T cells. Both IVIg and IL-11 have been shown to ameliorate experimental autoimmune encephalomyelitis (EAE), an animal model of MS. The objective of this study was to determine whether the protective effects of IVIg in EAE occur through an IL-11 and IL-11 receptor (IL-11R)-dependent mechanism.

**Methods:**

We measured IL-11 in the circulation of mice and IL-11 mRNA expression in various organs after IVIg treatment. We then followed with EAE studies to test the efficacy of IVIg in wild-type (WT) mice and in mice deficient for the IL-11 receptor (IL-11Rα^−/−^). Furthermore, we evaluated myelin-specific Th1 and Th17 responses and assessed spinal cord inflammation and demyelination in WT and IL-11Rα^−/−^ mice, with and without IVIg treatment. We also examined the direct effects of mouse recombinant IL-11 on the production of IL-17 by lymph node mononuclear cells.

**Results:**

IVIg treatment induced a dramatic surge (>1000-fold increase) in the levels of IL-11 in the circulation and a prominent increase of IL-11 mRNA expression in the liver. Furthermore, we found that IL-11Rα^−/−^ mice, unlike WT mice, although initially protected, were resistant to full protection by IVIg during EAE and developed disease with a similar incidence and severity as control-treated IL-11Rα^−/−^ mice, despite initially showing protection. We observed that Th17 cytokine production by myelin-reactive T cells in the draining lymph nodes was unaffected by IVIg in IL-11Rα^−/−^ mice, yet was downregulated in WT mice. Finally, IL-11 was shown to directly inhibit IL-17 production of lymph node cells in culture.

**Conclusion:**

These results implicate IL-11 as an important immune effector of IVIg in the prevention of Th17-mediated autoimmune inflammation during EAE.

## Introduction

Intravenous immunoglobulin (IVIg) is a blood-derived therapeutic prepared by pooling the immunoglobulin of thousands of donors [1], and is widely used to treat patients suffering from diseases such as primary immunodeficiency, Kawasaki disease, immune thrombocytopenia, Guillain-Barré syndrome, and chronic inflammatory demyelinating polyneuropathy [1]–[6]. In addition to these approved therapeutic uses, IVIg is also efficacious in many “off-label” clinical applications, particularly for autoimmune disorders such as myasthenia gravis and multiple sclerosis (MS) [7]–[9]. The unique ability of IVIg to provide therapeutic benefits for a wide variety of conditions has contributed to the increasing demand and costs of this blood product.

Currently, there is a lack of consensus as to the mechanism(s) underlying the immunomodulatory effects of IVIg [10]. Recent studies have indicated that the mechanism of IVIg may be independent of FcγRIIB [11]–[14] or antibody sialylation [15], [16]. This lack of an understanding of the molecular mechanism(s) of IVIg stands as a major hindrance to establishing treatment alternatives.

Multiple sclerosis (MS) is an autoimmune disease that is characterized by recurrent episodes of T cell-mediated immune attack on central nervous system (CNS) myelin, leading to axon damage and progressive disability [17]. Eighty-five percent of patients start with a relapsing-remitting form of disease (relapsing-remitting MS, RRMS) whereby they experience clinical episodes of neurological dysfunction, followed by periods of recovery [17]. It is in this recovery phase of the disease that immunomodulatory therapies (interferon-β, glatiramer acetate, natalizumab, and fingolimod) have efficacy in reducing relapse rates [18]. Although not a commonly used therapy for MS, intravenous immunoglobulin (IVIg) was shown in several clinical trials to reduce relapse rates and the number of brain lesions on MRI in patients with early RRMS [19]. IVIg is currently used in an “off-label” fashion to treat MS exacerbations, particularly in patients who are refractory to steroid treatment or who are pregnant and need safer treatment alternatives [20]. How IVIg exerts its clinical benefit in MS or other T cell-mediated autoimmune diseases is not completely understood. Various potential mechanisms have been proposed based on work done in the EAE model of MS: 1) circulating autoantibodies to myelin proteins may be targeted by IVIg; 2) IVIg can induce the expansion of regulatory T cells which can modulate the immune response in MS; 3) IVIg can downregulate pro-inflammatory cytokines such as IL-2, IFN-γ; 4) IVIg may prevent activated complement components from attaching to the surface of oligodendrocytes and myelin proteins [14], [21]–[24]. While each of these possible mechanisms has merit, there remain underexplored areas of understanding IVIg’s effects, such as through induction of specific immunomodulatory cytokines.

Interestingly, one microarray study identified interleukin-11 (IL-11) as amongst several immune-related genes that were upregulated following IVIg treatment in the T cells of MS patients [25]. IL-11 is a member of the gp130 cytokine family that is widely-expressed and has a range of biological activities including induction of hematopoiesis, regulation of bone resorption, and regulation of the liver response to injury [26], [27]. More recently, IL-11 was shown to have beneficial effects in the attenuation of EAE [28]. Taken together, these reports suggest that IL-11 is capable of ameliorating CNS autoimmune inflammation and further raise the possibility that this cytokine could be an immune effector of IVIg in the amelioration of EAE and MS.

The purpose of the present study was to test the hypothesis that IVIg-induced IL-11 and effects through the IL-11 receptor serve as a mechanism required by IVIg for optimal attenuation of the T cell-mediated CNS inflammatory response in EAE. Here we confirm that IL-11 is induced to high levels in the circulation of mice during EAE in response to IVIg treatment. Furthermore, we show that mice that are deficient in IL-11Rα are more resistant to the protective effects of IVIg treatment in preventing EAE and that this relates to a failure of IVIg to attenuate the CNS trafficking and IL-17A production by autoreactive T cells.

## Materials and Methods

### Ethics statement

All animal work was conducted according to relevant national and international guidelines. This study was carried out in strict accordance with the Policies and Guidelines of the Canadian Council on Animal Care and the Provincial Statute-Animals for Research Act. The work was done under protocol AUP 2764.1 which was approved by the University of Toronto-affiliated University Health Network Animal Care Committee.

### Animals

IL-11 receptor α-chain knockout (IL-11Rα^−/−^) or IL-11Rα^+/+^ (WT) littermate control females on the C57BL/6J background were from the Jackson laboratory (Bar Harbor, ME).

### EAE induction and IVIg treatment

Mice (8–10 wks of age) were immunized by subcutaneous injection of mice at two sites in the chest (50 µl/side) with an emulsion containing myelin oligodendrocyte glycoprotein amino acids 35–55 (MOG_35–55_) (2 mg/ml) (Stanford University Pan Facility, Stanford, CA) with Complete Freund’s Adjuvant (CFA) containing heat killed *Mycobacterium tuberculosis H37Ra* (4 mg/ml; Difco, Detroit, MI) [29]. Mice were also injected i.p. with 75 ng of pertussis toxin (List Biologicals, Campbell, CA) on the day of and 2 days after immunization. IVIg (Gammagard, Baxter Healthcare Corp., Toronto, ON; Privagen, CSL Behringer, Ottawa, ON, or Gammunex, Grifols, Los Angeles, CA) was administered daily (1 g/kg, i.p.) throughout, beginning on the day of immunization. Control groups received either sterile 1×PBS or an equivalent dose (1 g/kg) of human serum albumin (HSA; Canadian Blood Services, Toronto, ON). All mice were examined daily and were assessed for clinical scores of EAE as follows: 0 = no symptoms; 1 = tail paralysis; 2 = hindlimb or foot weakness; 3 = paralysis of one or both limbs; 4 = hindlimb paralysis and weakness in one or both forelimbs; 5 = moribund or dead [29].

### Assessment of MOG_35–55_ reactive T cell responses

The proliferation of and cytokine production by MOG_35–55_ reactive cells in spleen and lymph node mononuclear cell preparations were assessed at either day 10 or day 12 post-immunization with MOG_35–55_/CFA. Pertussis toxin was not administered in these studies to slow the egress of MOG_35–55_-reactive cells from the lymph nodes in order to more accurately capture Treg cell numbers in the peripheral immune compartment. For these studies, spleens and draining LNs were harvested from mice and were dissociated into a single cell suspension. After centrifugation (1200 rpm for 10 min), red blood cells were lysed using ACK lysis buffer (0.15 M ammonium chloride, 10 mM KHCO_3_, 0.1 mM Na_2_EDTA, pH. 7.4) and were washed in sterile 1×PBS. After a second centrifugation, lymph node and spleen mononuclear cells were resuspended in complete RPMI media: 2 mM L-glutamine, 1 mM sodium pyruvate, 0.1 mM nonessential amino acids, 100 U/ml, penicillin, 0.1 mg/ml streptomycin, 10% fetal calf serum (all from Life Technologies, Carlsbad, CA), and 50 µM 2-mercaptoethanol (Sigma, Oakville, ON). Cells were plated in triplicate in 96-well flat-bottomed plates (0.5×10^6^/well) with various concentrations of MOG_35–55_ (0–20 µg/ml) for 48–72 h. To measure the proliferation of MOG-reactive cells, cultures were pulsed at 72 h of culture with [H^3^]-thymidine (Perkin Elmer, Woodbridge, ON) and 18 h later incorporated [^3^H]-thymidine in counts per minute was determined. Cytokine levels in culture supernatants were measured using Ready-SET-Go ELISA kits and FlowCytomix assays (eBioscience, San Diego, CA) at 48 h (IL-2) or 72 h (TNF-α, IFN-γ and IL-17) of culture.

### Characterization of T cell compartment and flow cytometry

Spleens, lymph nodes (inguinal plus axillary), and thymi were harvested from age-matched female IL-11Rα^+/+^ and IL-11Rα^−/−^ mice and were dissociated into a single cell suspension. CNS mononuclear cells were isolated from spinal cords and cerebellums (pooled from multiple mice within each group) using collagenase digestion followed by Percoll gradient [29]. These cells were stained using the aqua live/dead stain (Invitrogen, Burlington, ON) and antibodies specific for the following cell surface markers (all from eBioscience): CD4 (GK1.5), CD8 (53–6.7), B220 (RA3-6B2), CD11c (N418), CD62L (MEL-14), CD44-PE (IM7) using standard protocols provided by eBioscience. CD4^+^CD25^+^FoxP3^+^ Treg were stained using reagents and antibodies provided with the FoxP3-staining kit (eBioscience) along with an antibody specific for ICOS (7E.17G9). Intracellular staining for IL-17A (eBio17B7) and IFN-γ (XMG1.2) was performed using antibodies from eBioscience and Perm/Wash buffer and staining protocols from BD Pharmingen. Data were acquired using the LSRII analyzer (BD Biosciences, Mississauga, ON) and were analyzed with FlowJo software (Treestar, Ashland, OR). Gates were set using fluorescence minus one controls.

### Measurement of IL-11 mRNA expression by quantitative reverse transcription-polymerase chain reaction (qRT-PCR)

Lymph nodes (inguinal and axillary), bone marrow, liver, and spleens were isolated from individual adult (8–10 week old) female C57BL/6J mice at 6 h after a single injection of IVIg (2.0 g/kg, i.p., Gammunex) or 1×PBS. Total RNA was isolated from these tissues using the Absolutely Total RNA kit (Agilent, Santa Clara, CA). First-strand cDNA synthesis from 2 µg of total RNA was carried out using the Superscript III kit (200 U, Invitrogen) according to the manufacturer’s directions, in a 20 µl reaction that also contained 200 ng oligo(dT)_18_ primer (ThermoScientific), 0.5 mM dNTPs, 1×first-strand buffer, and 5 mM DTT (all from Invitrogen). Primers used to amplify mouse cDNAs were synthesized by The Center for Applied Genomics (The Hospital for Sick Children, Toronto, Canada). Primer sequences were as follows: Mouse *il11*: forward primer (5′-CTGTGGGGACATGAACTGTG-3′), reverse primer (5′-CGTCAGCTGGGAATTTGTC-3′). Mouse *Actb*: forward primer (5′-GAGTCCGGCCCCTCCATCGT-3′), reverse primer (5′-GACTCAGGGCATGGACGCGA-3′). Real-time qRT-PCR reactions (25 µl) were conducted in duplicate using the Rotor-Gene RG-3000 thermocycler (Corbett Research, Montreal, Canada). Each reaction contained 25 ng template cDNA, 12.5 µL 2×SYBR Green PCR Master Mix (Applied Biosystems, Warrington, UK), 300 nM of each forward and reverse primer, and PCR grade H_2_O (Roche Diagnostics, Indianapolis, USA). Samples lacking reverse transcriptase (No RT) during first-strand cDNA synthesis served as a control for genomic contamination. Cycling parameters were as follows: initial denaturation at 95°C for 10 min followed by 40 cycles of amplification with 95°C for 15 seconds, 54°C (for *Actb*) or 58°C (for *il11*) for 30 seconds, and 60°C for 30 seconds. Biological replicates in the PBS or IVIg-treated groups (relative to beta-actin) were normalized to the average C_T_ of bone marrow samples taken from PBS-treated mice, by the comparative C_T_ method (also referred to as the 2^−ΔΔCT^).

### Measurement of circulating IL-11

IL-11 levels were measured in serum of mice using an ELISA kit (R&D systems, Inc., Minneapolis, MN).

### Histology

Spinal cords were harvested from EAE mice at the end-point of the experiment and were fixed in formalin and embedded in paraffin. Spinal cord transverse sections (10 sections/cord) were cut (4 µm thick) and were stained with Haematoxylin and Eosin and Luxol Fast Blue (Pathology Core, Toronto Center for Phenogenomics, Toronto, ON). Inflammation was largely in the spinal cord and the severity of this inflammation was scored as follows. Spinal cord sections (10 per mouse) were divided into four quadrants: the ventral funiculus, the dorsal funiculus and each lateral funiculus. Each observation of meningitis or the presence of perivascular immune cell infiltration in any of the quadrants was scored as 1 observation. Observations were then summed across all sections and the overall pathological score was expressed as the percentage of affected quadrants/total number of quadrants examined. To score demyelination in the spinal cord, the area of myelin pallor in each section of spinal cord was quantified using an image analysis program and attached microscope (Leica Application Suite, Leica Microsystems, Richmond Hill, ON) and was expressed as a percentage of total white matter area.

### Treatment of lymph node cells with mouse recombinant IL-11

Lymph nodes (axillary, cervical and inguinal) were harvested from C57BL6/J mice, were pooled, and were dissociated into a single cell suspension. Lymph node cells were resuspended in complete RPMI containing no added cytokines (Th0 conditions) or under Th17-skewing conditions (with added IL-6, TGF-β and anti-IFN-γ, all reagents from eBioscience) in the presence or absence of mouse recombinant IL-11 (R&D systems, Minneapolis, MN). Cells were then plated (0.5×10^6^/well) in 96-well flat-bottom plates that were pre-coated with anti-CD3 (145-2C11) and anti-CD28 (37.51) (both from eBioscience). The proliferation of, and cytokine productions by these cells were measured as described above.

## Results

### IVIg prevents the development of EAE

**Figure 1:**
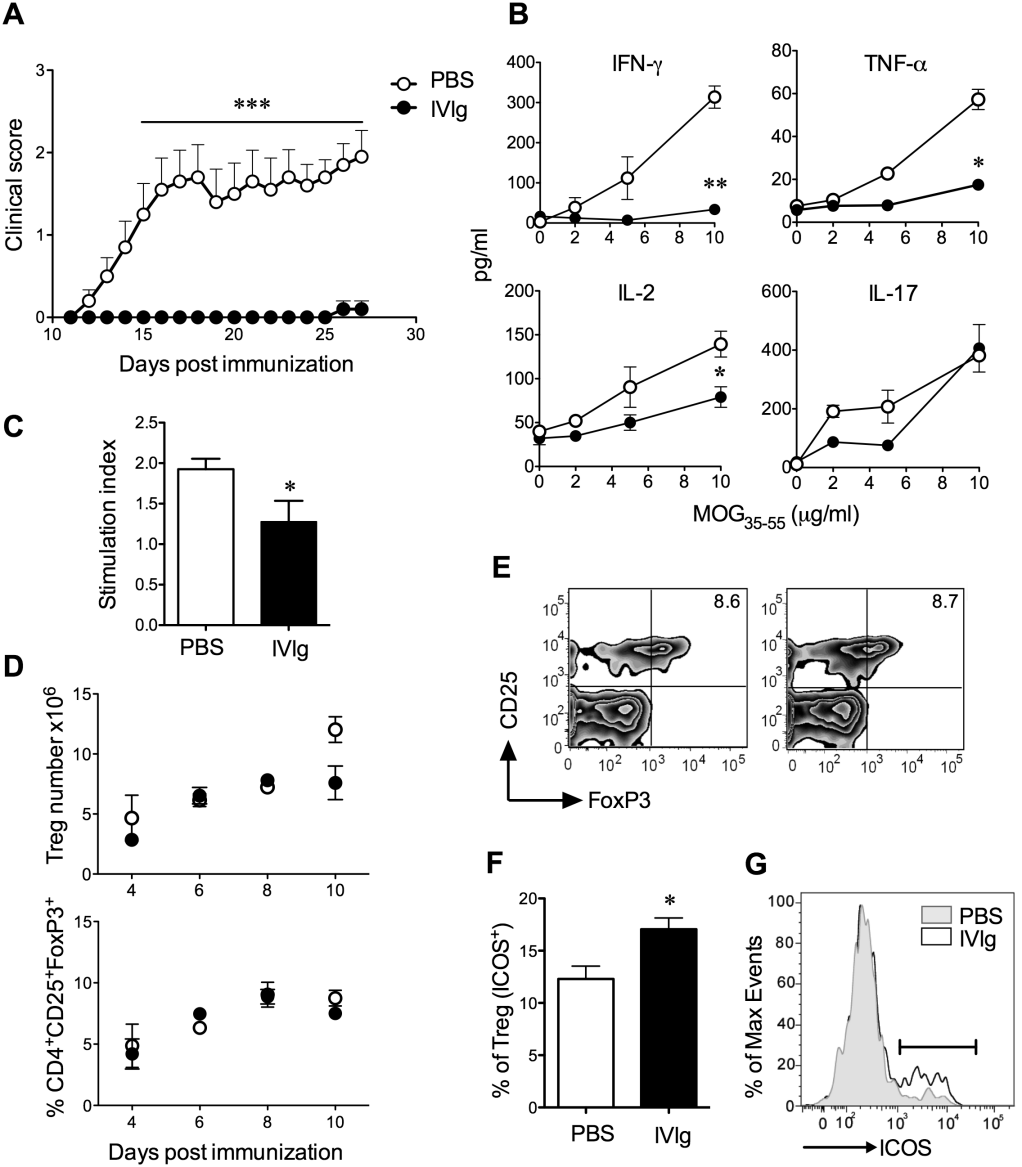
IVIg inhibits EAE by preventing the expansion and Th1 and Th17 cytokine production by MOG_35–55_ reactive T cells and by increasing ICOS expression by Tregs. EAE was induced in female C57BL/6J mice by immunization with MOG_35–55_ and CFA plus pertussis toxin and mice were administered daily i.p. injections of IVIg or 1×PBS beginning at day of disease induction (N = 12/group). At 10 post-immunization, spleens were collected from N = 2/mice per group and were pooled for ex vivo stimulation with MOG_35–55_. Remaining mice (N = 10/group) were followed for clinical signs. ***A***, shows the mean + SEM severity of clinical signs of mice in each group over the period of observation. ***B***, shows the levels of IL-2 (at 48 h), IFN-γ, TNF-α and IL-17A (at 72 h) in splenocyte cultures as measured by ELISA. Values are means + SEM of triplicate cultures in one representative experiment. ***C***, shows proliferation of cells in triplicate splenocyte cultures in response to MOG_35–55_ (5 µg/ml) was measured in counts per minute (cpm) and was expressed relative to the cpm in the non-peptide-containing wells. This ratio is the stimulation index. In ***A–C***, data are representative of 2–3 independent experiments. ***D–G***, represents a separate study where C57BL6/J mice were immunized with MOG_35–55_/CFA (without pertussis toxin) and were treated with PBS or IVIg daily. After 4, 6, 8, or 10 days, spleens were harvested, were processed into a single cell suspension for the determination of CD4, CD25, FoxP3, and ICOS using flow cytometry. ***D*** shows the frequency and number of CD4^+^CD25^+^FoxP3^+^ cells at these different time points after immunization. Values are means ± SEM 3 individual mice per group at each time point. ***E*** shows representative staining of FoxP3 and CD25 in the live, CD4^+^ gate while ***G*** shows representative ICOS staining of the live CD4^+^CD25^+^FoxP3^+^ population at day 6 post-immunization (peak of ICOS expression, data not shown). ***F*** Shows the mean ± SEM percent of Tregs that were positive for ICOS staining. Values are means + SEM percentages obtained in 3 individual mice per group. ***p<0.001, **p<0.01, *p<0.05 as determined using a t-test or Mann-Whitney U test.

We first aimed to characterize the immune mechanisms of IVIg treatment in MOG_35–55_/CFA-induced EAE in C57BL/6J mice. We induced this disease in females of this strain via immunization with MOG_35–55_ and CFA and then mice were administered daily injections of high-dose IVIg (1 g/kg) or 1×PBS as a control, starting on the day of disease induction. Similar to previous studies [14], [24], we observed that IVIg virtually prevented the development of EAE (Figure 1A); 100% of mice developed EAE in the control group and only 10% of mice developed clinical signs in the IVIg group.

To assess the underlying immune effects of IVIg treatment, we harvested the spleens of mice and examined the recall proliferation and cytokine responses of splenocyte mononuclear cells to MOG_35–55_ in culture. We observed that IVIg profoundly reduced the production of the T helper 1 (Th1) cytokines IFN-γ and TNF-α and moderately reduced the production of IL-2 and the Th17-associated cytokine IL-17A by MOG_35–55_-reactive T cells (Figure 1B). In addition, IVIg reduced the proliferation of splenocytes in response to MOG_35–55_ by almost half (Figure 1C). Thus, IVIg has an effect in dampening both Th1 and Th17 inflammatory pathways that are pathogenic in EAE and MS.

Recently, hypotheses on the mechanism of IVIg action have centred on the induction of CD4^+^CD25^+^FoxP3^+^ regulatory T cell (Treg) cells and an increase in their functionality [24], [30], [31]. To address whether IVIg induced an expansion of Tregs during EAE, we measured the frequency and number of these cells in the spleens of IVIg or PBS-treated mice at various time points after MOG_35–55_/CFA immunization. Although the frequency of Tregs tended to be elevated in the IVIg-treated group at 6 days post-immunization, this was not observed at other time points examined (Figure 1D, *bottom panel*). The numbers of CD4^+^CD25^+^FoxP3^+^ Tregs also were not different between PBS- and IVIg-treated groups (Figure 1D, *top panel*). We also examined the frequency of CD4^+^CD25^+^FoxP3^+^ Tregs that expressed Inducible T cell Co-Stimulator (ICOS), a protein that is important for Treg maintenance and suppressive functioning [32], [33]. We observed that a higher frequency of Tregs were positive for ICOS in the spleens of IVIg-treated as compared to PBS-treated mice (Figure 1F & G). Together, these results suggest that the dramatic effect of IVIg in preventing EAE in our hands is due to enhanced Treg function coupled with an inhibition of the expansion, trafficking, and Th1 and Th17 cytokine production by MOG_35–55_-reactive T cells.

### IVIg induces IL-11 production

**Figure 2:**
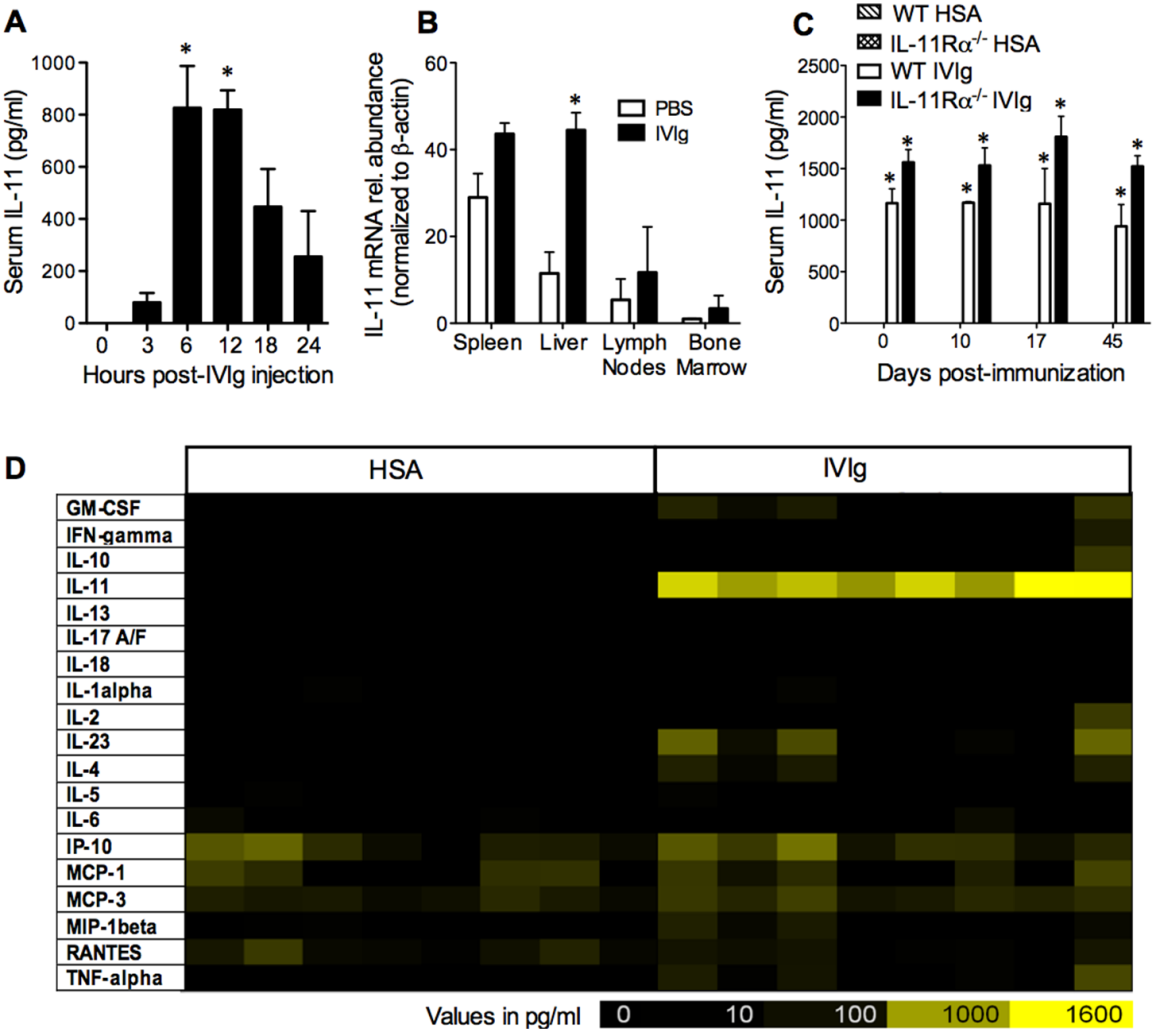
IVIg treatment induces a profound increase in the circulating levels of IL-11 in mice. ***A, B,*** Individual C57BL6/J mice were administered IVIg (2.0 g/kg) or PBS. ***A***, Serum was collected for IL-11 protein measurement using ELISA at 0, 3, 6, 12, 18 and 24 hours post-injection. *indicates a significant difference (*p<0.05) from time zero as determined using a one-way ANOVA and a Tukey post-hoc test. ***B***, Tissues (spleen, liver, bone marrow, and pooled axillary and inguinal lymph nodes) were collected from mice at 6 h post-injection, total RNA was isolated and was reverse-transcribed to cDNA, and IL-11 mRNAs were measured in samples using real-time qRT-PCR. Shown is the abundance of IL-11 mRNAs (normalized to beta-actin) and expressed as fold-change relative to the comparator sample (bone marrow sample from PBS-treated mouse). *indicates a difference from PBS-injected group as determined using a Student’s t-test assuming equal variances (two-tailed, *p<0.05). ***A & B*** show means + SEM of values obtained from individual mice. ***C***, EAE was induced in C57BL/6J mice with MOG_35–55_/CFA and pertussis toxin. Mice were injected with IVIg or HSA daily (1 g/kg, i.p.). Shown are the IL-11 levels (pg/ml) in the sera of mice at 6 h post-injection on the day of onset (0), and at 10, 17, and day 45 post-immunization with MOG_35–55_ and CFA. Values are means ± SEM of readings from 2–5 individual mice/group/time point. *indicates a significant difference (p<0.05) from the HSA-injected, genotype-matched counterpart. ***D***, shows a heat map of serum cytokine/chemokine levels taken at 6 h post IVIg or PBS injection with N = 8 mice/group. Each data box in the heat map represents a reading from an individual mouse.

Previous microarray studies of MS patient T cells indicated that IVIg upregulated the expression of six immune-related mRNAs (*xcl2, kir2ds1, map4k2, ptger4, casp2,* and *il11*) that included IL-11 [25]. To investigate whether IL-11 is produced at higher levels in mice with IVIg, we collected serum from C57BL/6J mice prior to IVIg and at various times post injection with IVIg (2.0 g/kg) and measured IL-11 levels by ELISA. Only negligible levels of IL-11 were detectable in the circulation of mice prior to IVIg administration (Figure 2A). However, after IVIg injection, we observed a dramatic increase in the levels of IL-11 that peaked between 6 and 12 hours post-injection (Figure 2A). Notably, when we compared this IL-11 response against the production of other cytokines and chemokines in the circulation, we found that IL-11 was the predominant cytokine induced by IVIg (Figure 2D).

To investigate the potential source of the IL-11, we conducted real-time qRT-PCR analysis of IL-11 mRNA expression in the spleen, bone marrow, lymph nodes, and liver of C57BL/6 mice at 6 h after a single i.p. injection of IVIg (2.0 g/kg) or PBS. Prior to IVIg, IL-11 mRNAs were most abundant in the spleen of PBS-injected mice (Figure 2B). However, upon IVIg treatment, IL-11 mRNA was only significantly increased in the liver relative to PBS-treated controls (Figure 2B); although, a trend for higher IL-11 mRNA expression was also observed in peripheral lymphoid organs. These findings suggest that the liver is the most likely source of the increased circulating IL-11 following IVIg administration.

Finally, we investigated the levels of IL-11 in the sera at 6 h post-IVIg injection at various time points throughout EAE progression. We found that the production of IL-11 was not detectable above background in any of the control (HSA)-injected mice, but was induced to an equivalent level by IVIg at all time points examined (Figure 2C, *open bars*).

### IVIg does not protect against EAE progression in IL-11Rα^−/−^ mice

**Figure 3:**
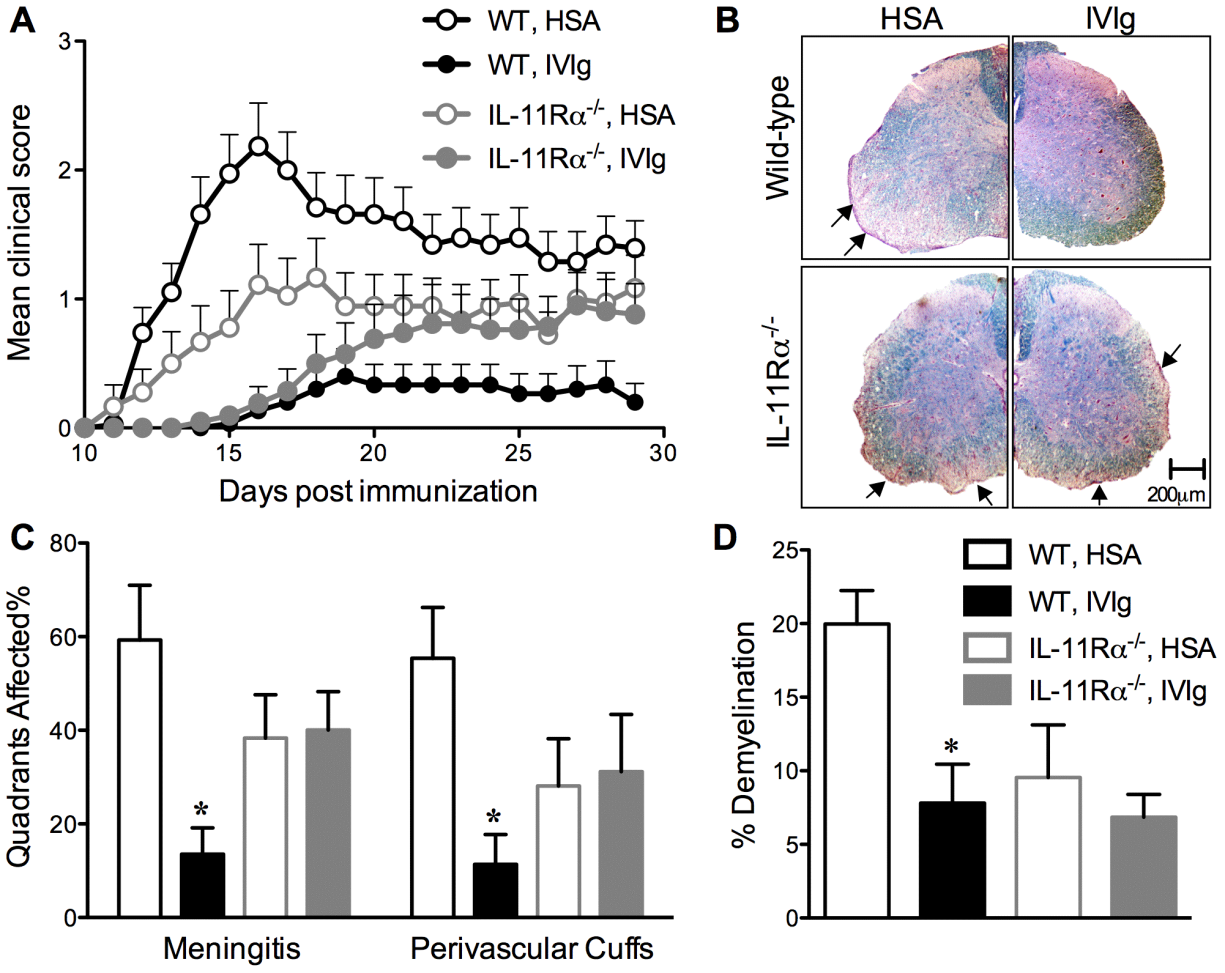
IL-11Rα^−/−^ mice are more resistant to the protective effects of IVIg treatment during EAE. EAE was induced in C57BL/6J IL-11Rα^+/+^ littermates (WT) or IL-11Rα^−/−^ mice that were administered IVIg or HSA (1 g/kg) daily beginning on the day of EAE induction. Mice were followed for clinical signs and a histological analysis of inflammation and demyelination was conducted at the end-point of the experiment. ***A***, shows the mean + SEM clinical scores of mice over a time-course of EAE. The graph shows combined data from three consecutive EAE studies that each contained (N = 5–10 mice/group). ***B***, shows representative spinal cord sections from control- or IVIg-treated IL-11Rα^−/−^ or WT mice stained with haematoxylin and eosin and luxol fast blue. ***C***, shows the percent quadrants of spinal cords (N = 10 sections/mouse) that were positive for meningitis or perivascular cuffs in EAE mice. ***D***, shows the percent demyelination in these spinal cord sections. Values in ***C & D*** are means + SEM of N = 4–6 mice per group for one representative experiment. *represents a significant difference (*p<0.05) from the HSA-treated, genotype-matched counterpart as determined by one-way ANOVA and a Tukey post-hoc test.

To address whether IVIg utilizes an IL-11-dependent mechanism to attenuate EAE, we compared the efficacy of IVIg to ameliorate EAE versus the control treatment, human serum albumin (HSA), in IL-11Rα^+/+^ (WT) and IL-11Rα^−/−^ mice. IL-11Rα^−/−^ mice display an overtly normal immune compartment (Table 1) and like WT mice, upregulate IL-11 upon IVIg treatment (Figure 2D). Upon induction of EAE, WT mice that received HSA treatment developed EAE with moderate severity and with high incidence while WT mice treated with IVIg had a greatly reduced incidence of disease (Figure 3A, Table 2). On the other hand, in IL-11Rα^−/−^ mice, EAE not only presented differently than in WT mice, but the resultant disease also responded differently to IVIg treatment. First, IL-11Rα^−/−^ mice displayed a reduced incidence of disease than WT mice resulting in lower median clinical scores (Table 2). Secondly, we observed that IL-11Rα^−/−^ mice were resistant to the protective effects of IVIg. Though IVIg had an equivalent effect in delaying the onset of EAE in IL-11Rα^+/+^ and IL-11Rα^−/−^ mice, IL-11Rα^−/−^ mice, unlike WT counterparts progressed to develop moderate clinical symptoms (Figure 3A, Table 2). The severity of these symptoms and the incidence of EAE also did not differ between HSA- and IVIg-treated IL-11Rα^−/−^ mice (Table 2). Although, the difference in the severity of acute EAE between IL-11Rα^−/−^ and WT mice made it difficult to compare the immunomodulatory effects of IVIg, our finding that this treatment was less effective in treating a relatively “milder” variant of EAE in IL-11Rα^−/−^ mice strongly suggests that having an intact IL-11R is required for mediating the full protective effects of IVIg in EAE.

**Table 1:** Characterization of the T Cell Compartment in IL-11Rα^+/+^ and IL-11Rα^−/−^ Mice.

	IL-11Rα^+/+^	IL-11Rα^−/−^
Body Weight	21.7 (0.3)	20.3 (0.5)
***Thymus***		
Weight (mg/kg bw)	2.4 (0.1)	2.3 (0.1)
Cellularity (×10^6^)	16.6 (1.8)	19.8 (1.9)
% CD4^+^CD8^+^	83.3 (1.5)	79.4 (1.4)
% CD4^+^	8.9 (0.7)	11.1 (0.9)
% CD8^+^	2.1 (0.2)	2.2 (0.3)
***Spleen***		
Weight (mg/kg bw)	5.0 (0.5)	5.4 (0.1)
Cellularity (×10^6^)	56.0 (2.3)	52.0 (7.0)
% CD4^+^	13.1 (1.1)	13.3 (0.6)
% CD4^+^ (CD25^+^FoxP3^+^)	7.8 (1.7)	8.4 (1.4)
% CD4^+^ (CD44^hi^)	17.8 (1.9)	18.4 (0.9)
% CD4^+^ (CD44^l^°CD62L^+^)	69.3 (2.2)	67.9 (1.1)
% CD8^+^	11.3 (1.4)	11.5 (0.6)
% B220^+^	55.7 (5.0)	55.4 (1.0)
% CD11c^+^	3.5 (0.6)	3.7 (1.2)
***Lymph Nodes***		
% CD4^+^	33.1 (1.4)	36.8 (1.1)
% CD4^+^ (CD25^+^FoxP3^+^)	11.6 (1.7)	13.0 (1.8)
% CD4^+^ (CD44^hi^)	11.1 (1.7)	8.6 (0.6)
% CD4^+^ (CD44^l^°CD62L^+^)	81.3 (0.9)	80.9 (0.9)
% CD8^+^	28.5 (0.7)	33.2 (2.6)
% B220^+^	32.9 (1.0)	30.2 (1.5)
% CD11c^+^	2.2 (0.2)	2.0 (0.2)

Shown are mean (SEM) of values obtained in 3–4 individual age-matched female mice. No measures were found to be significantly different (p<0.05) between IL-11Rα^+/+^ and IL-11Rα^−/−^ mice using a two-tailed Mann-Whitney U or T-test.

**Table 2:** Clinical Features of EAE in IL-11Rα^+/+^ and IL-11Rα^−/−^ mice treated with IVIg or HSA.

Group	N	Peak Score (EAE Cases)	Day of Onset (EAE Cases)	Cumulative Score	Percent Incidence
IL-11Rα^+/+^ HSA	19	3 (2)	13.3 (0.8)	27.4 (4.2)	84
IL-11Rα^+/+^ IVIg	16	1 (1)	16.8 (0.9)*	3.8 (1.7)*	27*
IL-11Rα^−/−^ HSA	18	2 (0.2)	14.8 (1.0)	16.0 (3.9)	67
IL-11Rα^−/−^ IVIg	21	2 (2)	19.6 (1.3)*	9.8 (3.0)	48

Values for day of onset, cumulative score are means (SEM). Values for peak score are shown as median (25 percentile). Between-group peak score was analyzed using a Kruskal Wallis test. The day of onset and cumulative score features were analyzed using a one-way ANOVA and Tukey post-hoc test. A chi-square test was used to analyze whether disease incidence differed between IVIg and HSA counterparts.

*indicates a significant difference (p<0.05) from the HSA counterpart. Sample sizes (N) are as indicated.

We also conducted a histopathological analysis of inflammation and demyelination in the spinal cords of mice at the end-point of the EAE experiment. WT mice treated with HSA exhibited the highest inflammation and demyelination scores, while IVIg treated WT mice showed considerably less inflammation and demyelination in the spinal cord (Figure 3B–D). Consistent with our finding of equivalent EAE severity in IL-11Rα^−/−^ mice treated with IVIg or the HSA, we observed no difference between these groups in the degree of inflammation or demyelination in the spinal cord (Figure 3B–D).

To gain insights into the differential effect of IVIg in EAE, we also assessed the frequency of IFN-γ- and IL-17A-producing CD4^+^ T cells in the CNS at the end-point of EAE. Spinal cord and cerebellum samples were harvested from 3–4 representative mice in each group, were pooled, and CNS mononuclear cells were isolated from these samples for flow cytometric staining. We observed that IL-11Rα^+/+^ mice treated with HSA displayed the highest frequency of CD4^+^ T cells in the CNS, with a majority of these cells producing IFN-γ and a minority of the cells producing IL-17A or co-producing IFN-γ and IL-17A (Figure S1). A similar T cell frequency and profile was observed in IL-11Rα^−/−^ mice treated with HSA (Figure S1). Coinciding with the lowered incidence of EAE, IVIg-treated IL-11Rα^+/+^ mice displayed a paucity of cytokine-producing CD4^+^ T cells in the CNS (Figure S1). On the other hand, IVIg-treated IL-11Rα^−/−^ mice displayed a comparatively higher CNS accumulation of CD4^+^ T cells that produced roughly equal amounts of IFN-γ and IL-17A (Figure S1). These findings suggest that IVIg treatment was less effective at inhibiting the trafficking of CD4^+^ T cells to the CNS in IL-11Rα^−/−^ mice.

### IVIg downregulates pro-inflammatory cytokine production in both wild-type and IL-11Rα^−/−^ mice, with the exception of IL-17A in the draining lymph nodes

**Figure 4:**
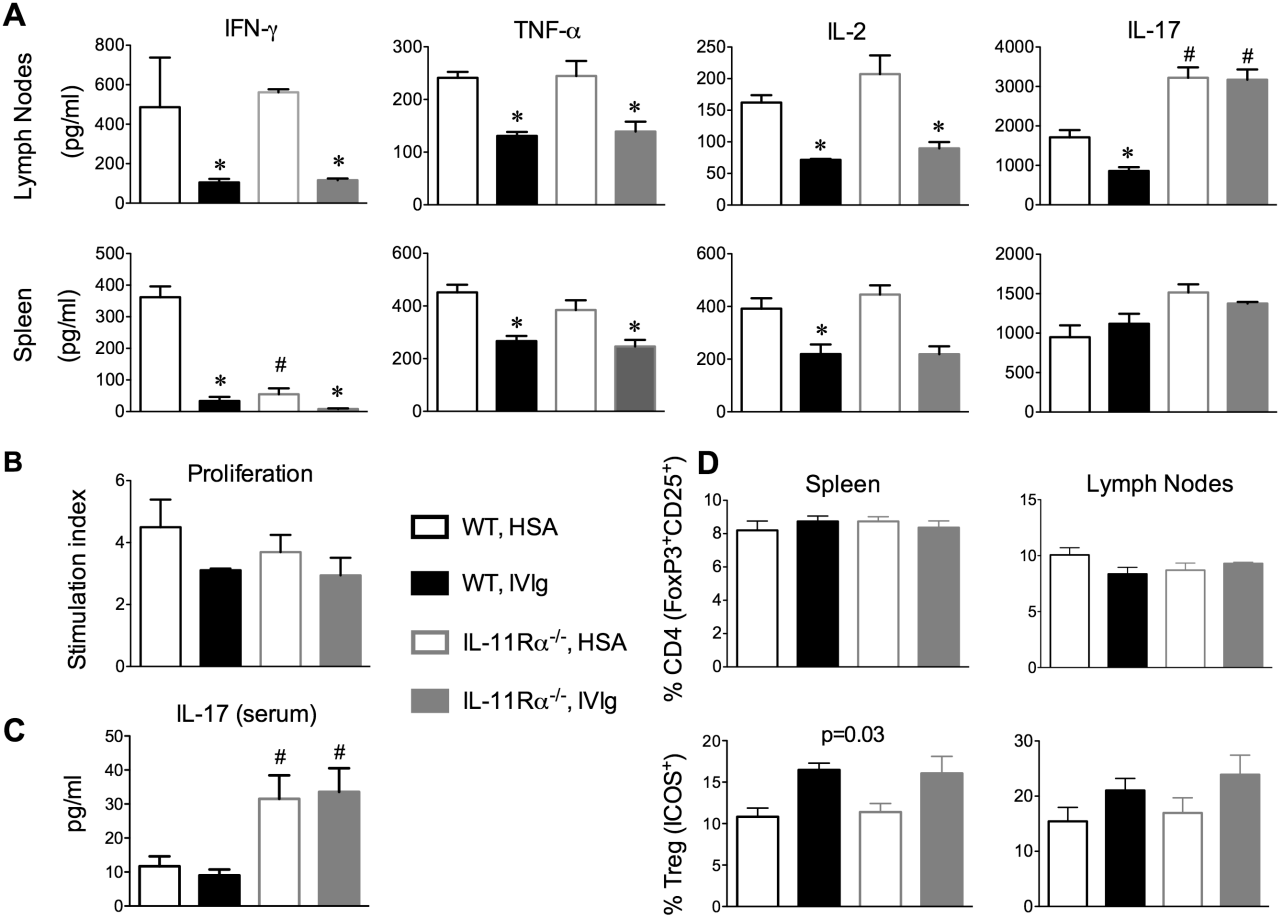
IVIg inhibits MOG_35–55_-induced pro-inflammatory cytokine production in IL-11Rα^−/−^ mice with the exception of IL-17A in the draining lymph nodes. IL-11Rα^+/+^ littermates (WT) and IL-11Rα^−/−^ mice were immunized with MOG_35–55_/CFA (no pertussis toxin), and were injected with either HSA or IVIg (1 g/kg, i.p.) daily. On day 6 (***D***) or on day 12 post-immunization (***A–C***), spleens and draining lymph nodes were collected, were processed into a single cell suspension, and were cultured with MOG_35–55_ (5 µg/ml). ***A***, The productions of IFN-γ, TNF-α, IL-2 and IL-17A were measured in supernatants of lymph node and splenocyte cultures of IVIg- or HSA-treated IL-11Rα^+/+^ (WT) and IL-11Rα^−/−^ mice. Values are means + SEM of triplicate cultures. These data are representative of three individual experiments. ***B***, The proliferation in counts per minute (cpm) of spleen cells was measured using a [H^3^]-thymidine incorporation assay. Shown is the stimulation index, which is the mean + SEM cpm in the MOG_35–55_-stimulated wells divided by the cpm in the media control wells. Values are representative of means + SEM of individual mice (N = 4–5/group) in one representative experiment. ***C***, Sera was taken from mice at 12 days post-immunization and the levels of IL-17 were measured using FlowCytomix assays. Values are means + SEM of individual mice (N = 4–5/group). ***D***, Lymph node and spleen mononuclear cells taken at day 6 post-immunization (peak of ICOS expression) were stained for CD4, CD25, FoxP3, and ICOS. The frequency of Tregs was determined, as was the frequency of these cells that were ICOS positive. Values are means + SEM of N = 3 individual mice per group. In all cases, groups were compared using a one-way ANOVA and Tukey post-hoc test. *p<0.05 indicates a difference of the IVig group from the HSA-treated, genotype-matched counterpart. #indicates a significant difference of the IL-11Rα^−/−^ group from the IL-11Rα^+/+^, treatment matched counterpart.

To gain further insights into the disparate effects of IVIg in IL-11Rα^+/+^ and IL-11Rα^−/−^ mice during EAE, we investigated the activity of MOG_35–55_-reactive T cells in the periphery of mice. We thus examined the proliferation and cytokine responses of mononuclear cells from the spleen and draining lymph node cells in response to stimulation with MOG_35–55_ *ex vivo*. The first thing we noted was that IL-11Rα^−/−^ mice develop a more Th17-biased immune response, with HSA-IL-11Rα^−/−^ mice exhibiting higher MOG_35–55_-induced IL-17A production and lower MOG_35–55_-induced IFN-γ production in the spleen as compared with IVIg-IL-11Rα^−/−^ counterparts (Figure 4A). We also detected higher levels of IL-17A in the circulation of IL-11Rα^−/−^ versus IL-11Rα^+/+^ mice (Figure 4C).

When contrasting the effects of IVIg in these mice, it was evident that IVIg was only effective at reducing the MOG_35–55_-elicited IL-17A production in the lymph nodes of WT, but not IL-11Rα^−/−^ mice (Figure 4A). On the other hand, IVIg induced an equivalent reduction in the proliferation and IL-2, IFN-γ, and TNF-α production by MOG_35–55_-reactive immune cells in these immune compartments in IL-11Rα^+/+^ and IL-11Rα^−/−^ mice (Figure 4A–B). We also compared the effects of IVIg on ICOS expression in Treg cells in the spleen and draining lymph nodes of these mice and observed a similar trend for enhanced ICOS expression with IVIg regardless of animal genotype (Figure 4D). Taken together, these data suggest that IVIg has dual effects in the inhibition of EAE, an effect in inhibiting IL-17A production or the CNS trafficking of CD4^+^ T cells that is dependent on the IL-11R and an effect in suppressing the proliferation or expansion of Th1 cells that is independent of the IL-11R. It was the defect in the MOG_35–55_-reactive Th17 responses that correlated with progression to EAE development in the IL-11Rα^−/−^ mice.

### IL-11 has direct effects in inhibiting IL-17A production by T cells

**Figure 5:**
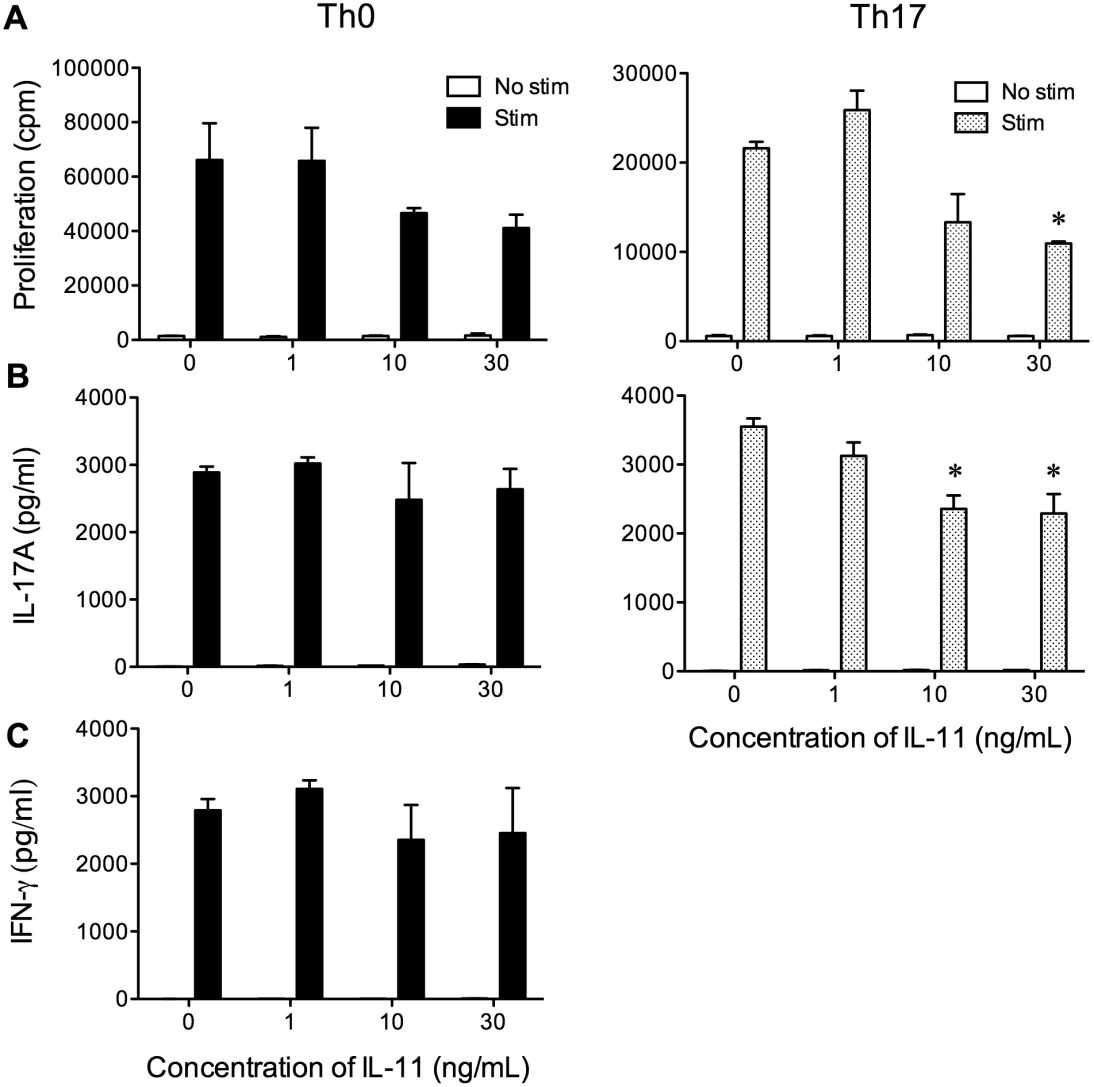
IL-11 inhibits the proliferation and IL-17A production by lymph node cells under Th17-polarizing conditions. Lymph node cells were harvested from C57BL/6J mice (N = 3/group). They were dissociated into a single-cell suspension and were stimulated with plate-bound anti-CD3 and anti-CD28 (0.5 µg/ml) in the absence of added cytokines (Th0) or in the presence of IL-6 (30 ng/ml), TGF-β (3 ng/ml) and anti-IFN-γ neutralizing antibody (10 µg/ml). The proliferation of (***A***) and IL-17A (***B***) and IFN-γ (***C***) cytokine production by lymph node cells was measured using H^3^-thymidine incorporation and ELISA assays, respectively. Values represent mean + SEM pg/ml or cpm in triplicate cultures. Results are representative of two individual experiments. *indicates a significant (p<0.05) difference from the 0 ng/ml concentration group as determined using a one-way ANOVA and Tukey post-hoc test. Note that IFN-γ was not detected under Th17 skewing conditions and is not shown.

Our finding that IL-11Rα^−/−^ mice exhibited Th17-biased inflammation in the periphery and were more resistant to IVIg suggested either that IVIg is less able to suppress Th17-mediated inflammation or that IL-11 and IL-11R signalling elicited by IVIg has the potential to attenuate Th17 responses. Our observation that IVIg reduced Th17 responses in WT mice supports more the latter possibility. To investigate whether exogenous IL-11 could suppress Th17 responses, we cultured lymph node cells *in vitro* with anti-CD3 and anti-CD28 in the presence of mouse recombinant IL-11 and measured the proliferation, IFN-γ, and IL-17A production by these cells. We observed that under Th0 conditions (no added cytokines), the addition of IL-11 to cultures had no significant effect on the proliferation, IFN-γ, or IL-17A production by these cells (Figure 5). However, under Th17-promoting conditions (with IL-6, TGF-β and anti-IFN-γ), IL-11 reduced the proliferation and IL-17A production of lymph node cells. Taken together, these data provide further evidence that Th17 cells may be an immune population that is affected by IL-11 in the IVIg-mediated attenuation of EAE.

## Discussion

IVIg has been shown in several clinical trials to reduce MS relapse rates and the number of brain lesions detected by MRI in patients with early relapsing-remitting MS [19]. This agent is currently used in an “off-label” fashion to treat MS exacerbations, particularly in patients who are refractory to steroid treatment or who are pregnant and need safer treatment alternatives [20]. How IVIg exerts its clinical benefit in MS is not known, however many potential mechanisms have been proposed [1], [9]–[16], [21]. The objective of this study was to test the hypothesis that IL-11 could be an immune effector of IVIg in the treatment of CNS autoimmunity in an animal model of MS. We show that IL-11 is the main cytokine upregulated in the serum of mice post-IVIg treatment and that IL-11 has an effect in attenuating Th17 cytokine production *in vitro*. Furthermore, mice that are deficient in the receptor for IL-11 are more resistant to IVIg-amelioration of EAE at later stages, correlating with an ineffectiveness of IVIg to inhibit IL-17A by MOG_35–55_-reactive T cells. Taken together, these results suggest that some of the effects of IVIg in the amelioration of EAE involve IL-11 actions in inhibiting Th17 responses.

Our finding that high-dose IVIg effectively prevented EAE development is in agreement with previous results in mouse and rat EAE models [14], [23], [24], [34], [35]. Similar to these studies, we also observed a profound effect of IVIg in the inhibition of the expansion of Th1 and Th17 cytokine production by myelin-reactive T cells [14]. Tregs have also been implicated as key effectors of IVIg therapy in murine models of autoimmune disease [24], [36] and in clinical studies [37]–[40]. In EAE, it was reported that IVIg induces a preferential expansion of these cells in the spleen [24]. While we observed a trend for an increased frequency of Tregs at 6 days post-immunization in the IVIg-treated group, this increase was not sustained at later time points that we examined. The reason for the discrepancy between our study and this previous report is not clear. However we speculate that given the anti-proliferative properties of IVIg, that the higher dose used in our study (1.0 g/kg) may have limited the expansion of Tregs that is reported to occur at the lower dose (0.8 g/kg) [14], [24]. Nonetheless, consistent with previous studies of enhanced Treg functioning post-IVIg [24], [30], [31], we observed enhanced Treg expression of ICOS, a molecule that is critical for the suppressive function of these cells [32], [33].

The finding that IL-11 was induced at very high levels in the serum of mice post-IVIg treatment is a novel observation and corresponds with one report of the induction of IL-11 mRNAs in IVIg-treated MS patient T cells [25]. Consistent with this report, we observed a tendency for IL-11 mRNAs to be elevated in peripheral lymphoid organs in response to IVIg treatment. However, the most prominent upregulation of IL-11 mRNA following IVIg treatment occurred in the liver. Coinciding with this observation, IL-11 is reported to be produced by the liver in response to tissue injury and oxidative stress and induces a compensatory proliferation of hepatocytes to mediate liver repair post-injury [27], [41]. IL-11 has also been shown to protect against the elevation of liver enzymes or TNF-α production in various models of acute liver injury [27], [42]–[44]. Given that a transient elevation in liver enzymes can occur in patients post-IVIg infusion [45], [46], it is possible that the surge in IL-11 evoked by IVIg is reflective of a stress response of the liver to this treatment.

Our studies in the IL-11Rα^−/−^ mice revealed an important role for IL-11 as an anti-inflammatory mediator of IVIg in the treatment of EAE. We found that IL-11Rα^−/−^ mice, though they developed a milder form of EAE, were more resistant to the protective effects of IVIg over time, and developed this disease with a similar severity and extent of CNS inflammation as HSA-treated counterparts. The major immune correlates of this resistance to IVIg protection was that this therapy failed to inhibit IL-17A production by MOG_35–55_ reactive T cells in the draining lymph nodes and was ineffective at inhibiting the CNS infiltration of Th1 and Th17 cells in IL-11Rα^−/−^ mice. Further establishing the link between IL-11 and inhibition of IL-17A, we showed that IL-11 inhibited the proliferation of, and IL-17A production by lymph node cells when cultured in the presence of IL-6 and TGFβ. The finding that IL-11 only attenuated IL-17A production under Th17-polarizing conditions suggests that IL-11 may interfere with IL-6- and TGF-β-dependent signals that lead to Th17 differentiation.

Our finding that IVIg was just as effective at attenuating the expansion of Th1 cells and delaying the onset of EAE in IL-11Rα^+/+^ and IL-11Rα^−/−^ mice clearly indicates that IVIg has protective effects in EAE that are independent of IL-11. In this respect, various other mechanisms have been proposed to explain the immune modulatory mechanisms of IVIg including modulation of the expression or function of FcγRs, induction of inhibitory cytokines, cytokine neutralization, scavenging of complement fragments, and the induction of T regulatory cells [21].

One finding that IL-11Rα^−/−^ mice developed a milder form of EAE than WT mice was somewhat unexpected, given the enhanced Th17 responses in the periphery in these mice and the pathogenic role of Th17 cells in the EAE model. This milder EAE could relate to the less robust Th1 responses observed in IL-11Rα^−/−^ mice. Indeed, during EAE, Th1 cells producing IFN-γ induce the production of CCL2 by microglia, which elicits the subsequent recruitment of CCR2^+^ inflammatory monocytes to the CNS [47]–[49]. It is this influx of inflammatory monocytes that precipitates the development of severe paralysis in this EAE model [48]. Furthermore, one study has shown that IFN-γ production is absolutely required for the entry of pathogenic Th17 cells into the CNS in this EAE model [50].

Our observation of milder EAE in IL-11Rα^−/−^ mice also conflicts with one previous report of the development of more acute EAE in this same mouse strain [28]. However, there were a number of differences in the methods of this other study that could have accounted for this different result: (1) they bred their mice in-house as opposed to purchasing them directly from Jackson laboratories, (2) they used males in their study instead of females, and (3) they used 10-fold higher doses of pertussis toxin to induce EAE. Regarding microbiota differences, it has been shown that certain pathogens if present (e.g., segmented filamentous bacterium) can bias the immune system towards the development of Th17 inflammation [51], which certainly could have interacted with IL-11 signalling to alter the severity of EAE. Regarding sex differences, it has been shown that the underlying inflammation that develops in mice during EAE is biased more towards Th1 in females [52]. Thus male IL-11Rα^−/−^ mice may have been less impacted by the reduced MOG_35–55_-specific Th1 responses. Although the pertussis toxin is an adjuvant with clear effects in enhancing the expansion and trafficking of Th1/Th17 cells [53]–[55], the much lower dose of adjuvant in our study relative to [28] is a less likely reason for the difference in our observations as the severity of EAE that developed in WT groups in each study was similar.

It is previously reported that IL-11 is expressed in the MS lesion by reactive astrocytes and that IL-11 can increase oligodendrocyte progenitor cell numbers, leading to an increase in the number of mature oligodendrocytes [56]. More recently, it was shown that overexpression of IL-11 in the brain reduces the extent of demyelination and enhances remyelination in the cuprizone-induced demyelinating disease model [57]. We did not observe histologic evidence of more severe demyelination in IL-11Rα^−/−^ mice during EAE. However this is not unexpected given that the acute inflammatory response that triggers demyelination in this model was less severe in IL-11Rα^−/−^ versus WT mice. Our studies therefore do not discount a role for IL-11 in myelin protection during EAE. Future studies should evaluate whether the IVIg-induction of IL-11 has a consequence on myelination and repair in a non-autoimmune-based demyelination/remyelination model.

In conclusion, our results suggest a novel mechanism involving IL-11 in IVIg-induced immunomodulation. Future studies will address whether this mechanism also operates in IVIg protection in other models of autoimmune disease. A limitation of our current study is that we examined the effects of IVIg exclusively as a preventative therapy for EAE and did not examine its potential benefits as a treatment therapy, particularly in respect to possible differences in outcome when using IL-11Rα^−/−^ mice. We nevertheless have made some important observations regarding the mechanism of IVIg amelioration in the mouse model of MS. Our findings implicate IL-11 as an important immune effector of IVIg therapy, which is a novel finding that may help to unravel the mechanism of action of IVIg, which has been elusive for more than three decades. Our findings should also prove useful in future studies using the EAE mouse model and in human therapies that utilize IVIg.

## Supporting Information

Figure S1 **EAE was induced in IL-11Rα+/+ and IL-11Rα−/− mice via immunization with MOG p35–55/CFA.** At the end point of the experiment (30 d post-immunization), mice with representative scores of the group (N = 3–5) were sacrificed, and spinal cords and brains were harvested. CNS mononuclear cells were isolated from spinal cords and cerebellums (that were pooled from multiple mice within each group) using collagenase digestion followed by Percoll gradient. These cells were stained with aqua live/dead stain and CD4-PE-Cy5, IFN-γ-APC, and IL-17-PE antibodies and were analyzed with flow cytometry. The top panel shows CD4 by FSC staining in the live gate. The box shows the percentage of CD4+ T cells in each group. The bottom panel shows IFN-γ and IL-17A staining in the CD4+ gate for each group.
(DOCX)
