# Correction: Optimal Attenuation of Experimental Autoimmune Encephalomyelitis by Intravenous Immunoglobulin Requires an Intact Interleukin-11 Receptor

**DOI:** 10.1371/journal.pone.0113001

**Published:** 2014-11-04

**Authors:** The PLOS ONE Staff

There are errors in the author affiliations. The affiliations should appear as shown here:

Carlyn A. Figueiredo^1,2^, Paulina C. Drohomyrecky^4^, Stephen D. S. McCarthy^1,2^, Danila Leontyev^1^, Xue-Zhong Ma^1^, Donald R. Branch^1,2,3*☯^, Shannon E. Dunn^3,4,5*☯^

1 Centre for Innovation, Canadian Blood Services, 67 College Toronto, Toronto, Ontario, Canada,

2 Department of Laboratory Medicine and Pathobiology, University of Toronto, Toronto, Ontario, Canada,

3 Toronto General Research Institute, University Health Network, Toronto, Ontario, Canada,

4 Department of Immunology, University of Toronto, Toronto, Ontario, Canada,

5 Women’s College Research Institute, Toronto, Ontario, Canada

* Email: don.branch@utoronto.ca (DRB); sdunn@uhnresearch.ca (SED)

These authors contributed equally to this work.
