# Supplementary material for: Optimal Attenuation of Experimental Autoimmune Encephalomyelitis by Intravenous Immunoglobulin Requires an Intact Interleukin-11 Receptor
Source: PLoS One. 2014 Jul 31;9(7):e101947. doi: 10.1371/journal.pone.0101947 (PMC4117465; doi:10.1371/journal.pone.0101947)
Supplement: Figure S1 — EAE was induced in IL-11Rα+/+ and IL-11Rα−/− mice via immunization with MOG p35–55/CFA. At the end point of the experiment (30 d post-immunization), mice with representative scores of the group (N = 3–5) were sacrificed, and spinal cords and brains were harvested. CNS mononuclear cells were isolated from spinal cords and cerebellums (that were pooled from multiple mice within each group) using collagenase digestion followed by Percoll gradient. These cells were stained with aqua live/dead stain and CD4-PE-Cy5, IFN-γ-APC, and IL-17-PE antibodies and were analyzed with flow cytometry. The top panel shows CD4 by FSC staining in the live gate. The box shows the percentage of CD4+ T cells in each group. The bottom panel shows IFN-γ and IL-17A staining in the CD4+ gate for each group. (DOCX) [file pone.0101947.s001.docx]

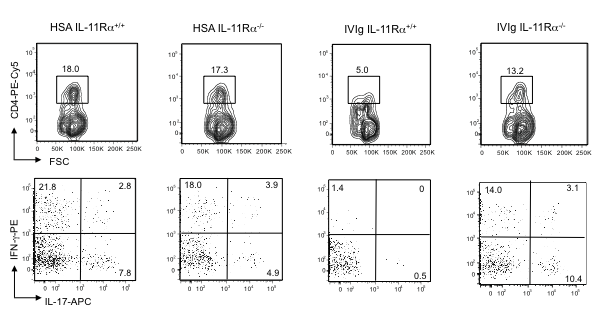


**Figure S1.** EAE was induced in IL-11Rα^+/+^ and IL-11Rα^-/-^ mice via immunization with MOG p35-55/CFA. At the end point of the experiment (30 d post-immunization), mice with representative scores of the group (N=3-5) were sacrificed, and spinal cords and brains were harvested. CNS mononuclear cells were isolated from spinal cords and cerebellums (that were pooled from multiple mice within each group) using collagenase digestion followed by Percoll gradient. These cells were stained with aqua live/dead stain and CD4-PE-Cy5, IFN-γ-APC, and IL-17-PE antibodies and were analyzed with flow cytometry. The top panel shows CD4 by FSC staining in the live gate. The box shows the percentage of CD4^+^ T cells in each group. The bottom panel shows IFN-γ and IL-17A staining in the CD4^+^ gate for each group.
